# β_2_-Adrenergic Receptor Gene Polymorphisms Are Associated with Cardiovascular Events But not All-Cause Mortality in Coronary Artery Disease Patients: A Meta-Analysis of Prospective Studies

**DOI:** 10.1089/gtmb.2018.0153

**Published:** 2019-02-08

**Authors:** Yanrong Li, Huiping Yuan, Liang Sun, Qi Zhou, Fan Yang, Ze Yang, Deping Liu

**Affiliations:** ^1^ Department of Cardiology, Beijing Hospital, National Center of Gerontology, Beijing, People's Republic of China.; ^2^ The MOH Key Laboratory of Geriatrics, Beijing Hospital, National Center of Gerontology, Beijing, People's Republic of China.

**Keywords:** β-adrenergic receptor, polymorphism, coronary artery disease, meta-analysis

## Abstract

***Aims:*** β-Adrenergic receptors (ADRBs) play a pivotal role in cardiovascular disease. Recently, genetic polymorphisms of *ADRB1* and *ADRB2* have been suggested to be associated with cardiovascular events and all-cause mortality in coronary artery disease (CAD) patients, but the results of relevant studies are inconsistent and controversial. Therefore, we performed a meta-analysis to investigate the association between *ADRB1* and *ADRB2* polymorphisms with cardiovascular events and all-cause mortality in CAD patients.

***Materials and Methods:*** The PubMed, Ovid, EMBASE, Cochrane, and CINAHL databases were searched for eligible studies published before April 2018. A total of 5495 patients from eight studies were included in our meta-analysis.

***Results:*** We found that CAD patients harboring the *ADRB2* rs1042714 Glu27 allele exhibited a positive association with cardiovascular events (risk ratio [RR] = 1.31, 95% confidence interval [CI]: 1.08–1.58, *p* = 0.006), but not with all-cause mortality (RR = 0.97, 95% CI: 0.70–1.35, *p* = 0.859), compared with patients who were Gln27 homozygotes. No other significant associations were observed between *ADRB1* (rs1801252, rs1801253), *ADRB2* (rs1042713, rs1800888) polymorphisms and cardiovascular events or all-cause mortality in CAD patients.

***Conclusion:*** This study suggests that the identified *ADRB2* polymorphism could influence the outcomes of CAD patients, showing important clinical value.

## Introduction

Coronary artery disease (CAD) is one of the leading causes of disease in developed and developing countries. The β_1_-adrenergic receptor (ADRB1) and β_2_-adrenergic receptor (ADRB2) play a pivotal role in the regulation of the cardiovascular system (Dhein *et al.*, 2017; Xia *et al.*, 2017). There are two major single nucleotide polymorphisms (SNPs) in the *ADRB1* gene: the Ser49Gly (rs1801252) and Arg389Gly (rs1801253) polymorphisms; the *ADRB2* gene exhibits three major SNPs: the Arg16Gly (rs1042713), Gln27Glu (rs1042714), and Thr164Ile (rs1800888) polymorphisms.

Several studies have examined the influence of the *ADRB1* and *ADRB2* polymorphisms on cardiovascular events and all-cause mortality in CAD patients. For example, Zaugg *et al.* (2007) observed that carriers of at least one Gly allele of the *ADRB1* rs1801253 polymorphism showed a greater number of cardiovascular adverse events than Arg homozygotes among CAD patients. However, Li *et al.* (2013) found no relationship between the *ADRB1* rs1801253, *ADRB2* rs1042713, and *ADRB2* rs1042714 polymorphisms and cardiovascular events in Han Chinese patients with CAD.

To better understand the interactions between the *ADRB1* (rs1801252 and rs1801253) and *ADRB2* (rs1042713, rs1042714, and rs1800888) polymorphisms and cardiovascular events as well as all-cause mortality in CAD patients, we undertook a meta-analysis with the aim of obtaining information for individual CAD prognostication and potential clinical application.

## Materials and Methods

### Search strategy

We carried out a comprehensive search of electronic databases, including PubMed, Ovid, EMBASE, Cochrane, and CINAHL, to identify relevant publications reporting an association between *ADRB1* and *ADRB2* polymorphisms and cardiovascular events as well as all-cause mortality in CAD patients, with the most recent publication date being April 2018. We used the search terms “coronary artery disease (CAD)” or “coronary heart disease (CHD)” or “ischemic heart disease (IHD)” or “myocardial infarction (MI)” or “acute coronary syndrome (ACS)” or “angina pectoris” or “atherosclerosis” or “ASCVD” and “β adrenergic receptor” or “beta adrenergic receptor” or “ADRB” in combination with “polymorphism” or “variation” or “variant” or “allele” or “mutation” or “SNP.” Additional relevant publications were identified through manual searches of the bibliographies of the retrieved studies and recent reviews.

Studies that met the following criteria were included: (1) prospective study design with patients who underwent follow-up for more than 1 year; (2) investigation of the association between *ADRB1* and *ADRB2* polymorphisms and cardiovascular events or all-cause mortality in CAD patients among unrelated subjects; (3) diagnosis of CAD based on previous myocardial infarction (MI), percutaneous coronary intervention (PCI), coronary artery bypass grafting, angiographic evidence, or angina patients with a positive stress test; and (4) the primary outcomes were cardiovascular events or all-cause mortality, wherein cardiovascular events included death, cardiac death, MI, heart failure, unstable angina, coronary revascularization, cardiac hospitalization, stroke, and cerebrovascular insult. Two investigators (Y.L. and H.Y.) screened all eligible studies independently. Any disagreements between the two investigators were resolved through discussion.

### Data extraction

Data were extracted from all eligible studies by primary investigators using a standardized extraction form. The following information was collected: first author's name, publication year, country, ethnicity of the population studied, age, gender, sample size, polymorphisms, outcomes, duration of follow-up, genotyping methods, Hardy–Weinberg equilibrium (HWE) in controls, quality scores, cases and controls with wild and variant genotypes, risk ratios (RRs), and 95% confidence intervals (95% CIs) of cardiovascular events or all-cause mortality. If any of this information was not provided in the publication, the authors were contacted via e-mail for more detailed data.

### Quality assessment

The quality of the identified studies was assessed according to a “methodological quality assessment scale,” which was modified from the study by Yuan *et al.* (2016). Five items, including the representativeness of the cases, the source of controls, sample size, quality control of the genotyping methods, and HWE, were evaluated on this scale. The quality scores ranged from 0 to 10, with a high score indicating a good-quality study.

### Statistical methods

The dominant models were evaluated for the association of *ADRB1* and *ADRB2* polymorphisms with cardiovascular events and all-cause mortality in CAD patients because there were insufficient data for specific cases and controls according to genotype in a few studies. The distribution of the genotypes in the control group was tested for HWE, where *p* < 0.05 was considered to indicate that the distribution of genotypes in the control group deviated from HWE. RRs and 95% CIs were calculated for the cardiovascular events and all-cause mortality in CAD patients. Statistical heterogeneity between eligible studies was evaluated by using the Cochran's Q statistic and *I*^2^ test. *p* < 0.1 indicated substantial heterogeneity across studies, and a random-effects model was chosen to perform analyses; otherwise, a fixed-effects model was selected. Sensitivity analyses were conducted to evaluate the stability of the results. The leave-one-out method was used to evaluate each study, and a pooled estimate was calculated for the remaining studies. Begg's funnel plots were generated to qualitatively evaluate publication bias; Egger's test was performed to quantitatively assess the publication bias. All *p* values were two sided. All statistical analyses were performed using STATA software version 11.0 (STATA Corporation, College Station, TX).

## Results

### Study characteristics

**FIG. 1.**
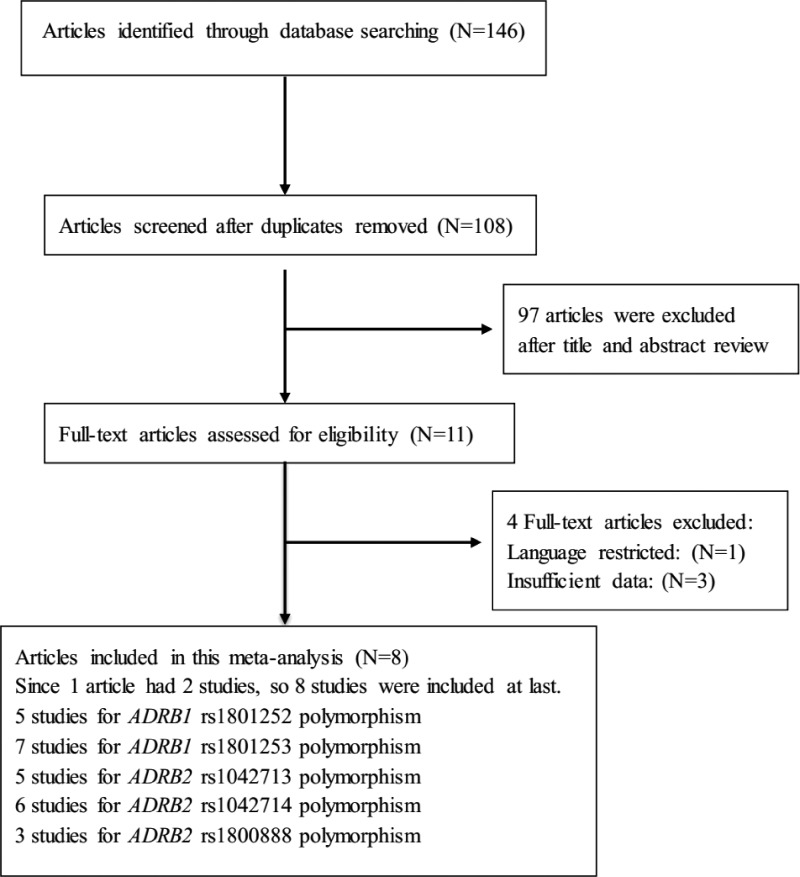
Flow diagram of study identification. *ADRB1*, β_1_-adrenergic receptor gene; *ADRB2*, β_2_-adrenergic receptor gene.

The process of literature retrieval and exclusion is shown in Figure 1. Eight studies were included in our meta-analysis (Lanfear *et al.*, 2005; Zaugg *et al.*, 2007; Pacanowski *et al.*, 2008; Piscione *et al.*, 2008; Tseng *et al.*, 2008; Li *et al.*, 2013; Feldman *et al.*, 2015). Women with obstructive CAD (Pacanowski *et al.*, 2008), the Heart and Estrogen Replacement Study cohort (Tseng *et al.*, 2008), and CAD patients who were treated with β-blockers (Lanfear *et al.*, 2005) were enrolled in this meta-analysis. Since two cohorts of patients were enrolled in the study by Feldman *et al.* (2015), the two cohorts were considered as independent studies. A total of 5495 patients from eight studies were included in our meta-analysis. The detailed characteristics of eligible studies included in our meta-analysis are shown in Table 1. In the study by Zaugg *et al.* (2007), deviation from HWE was found for the *ADRB1* rs1801253 polymorphism; therefore, we excluded this study from the analysis of the association with cardiovascular events in CAD patients. The specific wild-type/variant patients and the associations of *ADRB1* and *ADRB2* polymorphisms with clinical outcomes according to the eligible studies are shown in Table 2.

**Table 1.** Characteristics of Studies Included in the Meta-Analysis

*First author (year)*	*Country*	*Ethnicity*	*Age (years)*	*Sex (male%)*	*Sample size*	*Polymorphisms*	*Outcomes*	*Follow-up (years)*	*Method*	*HWE*	*Quality score*
Zaugg (2007)	Switzerland	Caucasian	NA	NA	189	rs1801252, rs1042713, rs1042714	Cardiovascular events	1	TaqMan assay	Yes	8
Li (2013)	China	Han Chinese	NA	NA	545	rs1801253, rs1042714, rs1042714	Cardiovascular events	1	Sequenom	Yes	7
Pacanowski (2008)	USA	NA	62 ± 12	0	227	rs1801253	Cardiovascular events, All-cause mortality	5.8	PCR and single-primer extension, PCR and luciferase-based assays	Yes	8
Tseng (2008)	USA	Caucasian, African American, Hispanic	NA	0	2223	rs1801252, rs1801253, rs1042713, rs1042714	All-cause mortality	6.8	Sequenom	Yes	7
Lanfear (2005)	USA	Caucasian, African-American	60 (12)	64	735	rs1801252, rs1801253, rs1042714	All-cause mortality	3	Applied Biosystems genotyping assays	Yes	7
Feldman 1 (2015)	USA	Caucasian	61.2 (54.8, 69.1)	86.5	532	rs1801252, rs1801253, rs1042713, rs1042714, rs1800888	Cardiovascular events, All-cause mortality	NA	PCR-RFLP	NA	8
Feldman 2 (2015)	USA	Caucasian	62.1 (55.2, 69.3)	86.4	714	rs1801252, rs1801253, rs1042713, rs1042714, rs1800888	Cardiovascular events, All-cause mortality	NA	PCR-RFLP	NA	8
Piscione (2008)	Italy	NA	NA	NA	330	rs1800888	Cardiovascular events	3 ± 0.33	PCR-RFLP	NA	5

Since two cohorts of patients were enrolled in the study by Feldman *et al.* (2015), the two cohorts were considered as independent studies (Feldman 1 and Feldman 2).

HWE, Hardy–Weinberg equilibrium; NA, not available; PCR, polymerase chain reaction; PCR-RFLP, polymerase chain reaction-restriction fragment length polymorphism.

**Table 2.** Association of *ADRB1* and *ADRB2* Gene Polymorphisms with Clinical Outcomes According to Eligible Studies

*Polymorphism*	*Outcome*	*First author (year)*	*Wild type,* n *(cases/controls)*	*Variants,* n *(cases/controls)*	*RR*	*95% CI*	p
rs1801252	Cardiovascular events	Feldman 1 (2015)	305/112	74/41	0.90	0.70–1.16	0.404
		Feldman 2 (2015)	338/221	95/59	1.02	0.81–1.28	0.874
		Zaugg (2007)	NA	NA	1.57	0.82–2.98	0.170
	All-cause mortality	Feldman 1 (2015)	172/245	43/72	0.97	0.69–1.36	0.861
		Feldman 2 (2015)	162/397	46/108	1.02	0.74–1.42	0.893
		Tseng (2008)	NA	NA	0.85	0.63–1.15	0.300
		Lanfear (2005)	49/360	18/129	1.02	0.58–1.82	0.933
rs1801253	Cardiovascular events	Li (2013)	23/276	21/196	1.05	0.54–2.02	0.887
		Feldman 1 (2015)	198/98	180/55	1.23	1.00–1.50	0.046
		Feldman 2 (2015)	227/141	206/140	0.94	0.78–1.13	0.511
		Pacanowski (2008)	35/71	44/77	1.16	0.67–2.01	0.598
	All-cause mortality	Feldman 1 (2015)	110/186	104/131	1.16	0.89–1.52	0.282
		Feldman 2 (2015)	112/256	96/250	0.93	0.71–1.22	0.588
		Tseng (2008)	NA	NA	0.96	0.75–1.23	0.750
		Pacanowski (2008)	14/92	24/97	1.63	0.79–3.33	0.182
		Lanfear (2005)	38/253	29/222	0.87	0.52–1.46	0.596
rs1042713	Cardiovascular events	Li (2013)	14/185	27/272	0.81	0.37–1.77	0.599
		Feldman 1 (2015)	60/22	319/131	0.94	0.71–1.24	0.661
		Feldman 2 (2015)	56/51	377/230	1.25	0.95–1.66	0.116
	All-cause mortality	Feldman 1 (2015)	37/45	178/272	0.82	0.58–1.17	0.281
		Feldman 2 (2015)	28/79	180/427	1.08	0.73–1.61	0.702
		Tseng (2008)	NA	NA	1.33	0.91–1.92	0.880
		Lanfear (2005)	82/327	15/132	2.21	1.23–3.97	0.01
rs1042714	Cardiovascular events	Li (2013)	32/378	12/112	1.66	0.81–3.42	0.166
		Feldman 1 (2015)	135/51	244/102	0.88	0.72–1.09	0.248
		Feldman 2 (2015)	125/105	308/176	1.27	1.03–1.56	0.116
		Zaugg (2007)	NA	NA	1.40	0.73–2.66	0.310
	All-cause mortality	Feldman 1 (2015)	82/104	133/213	0.81	0.62–1.07	0.139
		Feldman 2 (2015)	56/174	152/332	1.29	0.95–1.75	0.106
		Tseng (2008)	NA	NA	1.27	0.97–1.67	0.086
		Lanfear (2005)	36/188	34/308	0.58	0.35–0.95	0.030
rs1800888	Cardiovascular events	Feldman 1 (2015)	368/150	10/3	1.14	0.61–2.14	0.679
		Feldman 2 (2015)	420/273	13/7	1.02	0.59–1.78	0.937
		Piscione (2008)	NA	NA	4.10	1.95–8.64	0.0001
	All-cause mortality	Feldman 1 (2015)	209/309	5/8	1.19	0.49–2.89	0.699
		Feldman 2 (2015)	200/493	8/12	1.47	0.73–2.99	0.282

95% CI, 95% confidence interval; *ADRB1*, β_1_-adrenergic receptor gene; *ADRB2*, β_1_-adrenergic receptor gene; NA, not available; RR, risk ratio.

### Association of *ADRB1* and *ADRB2* polymorphisms with cardiovascular events

**FIG. 2.**
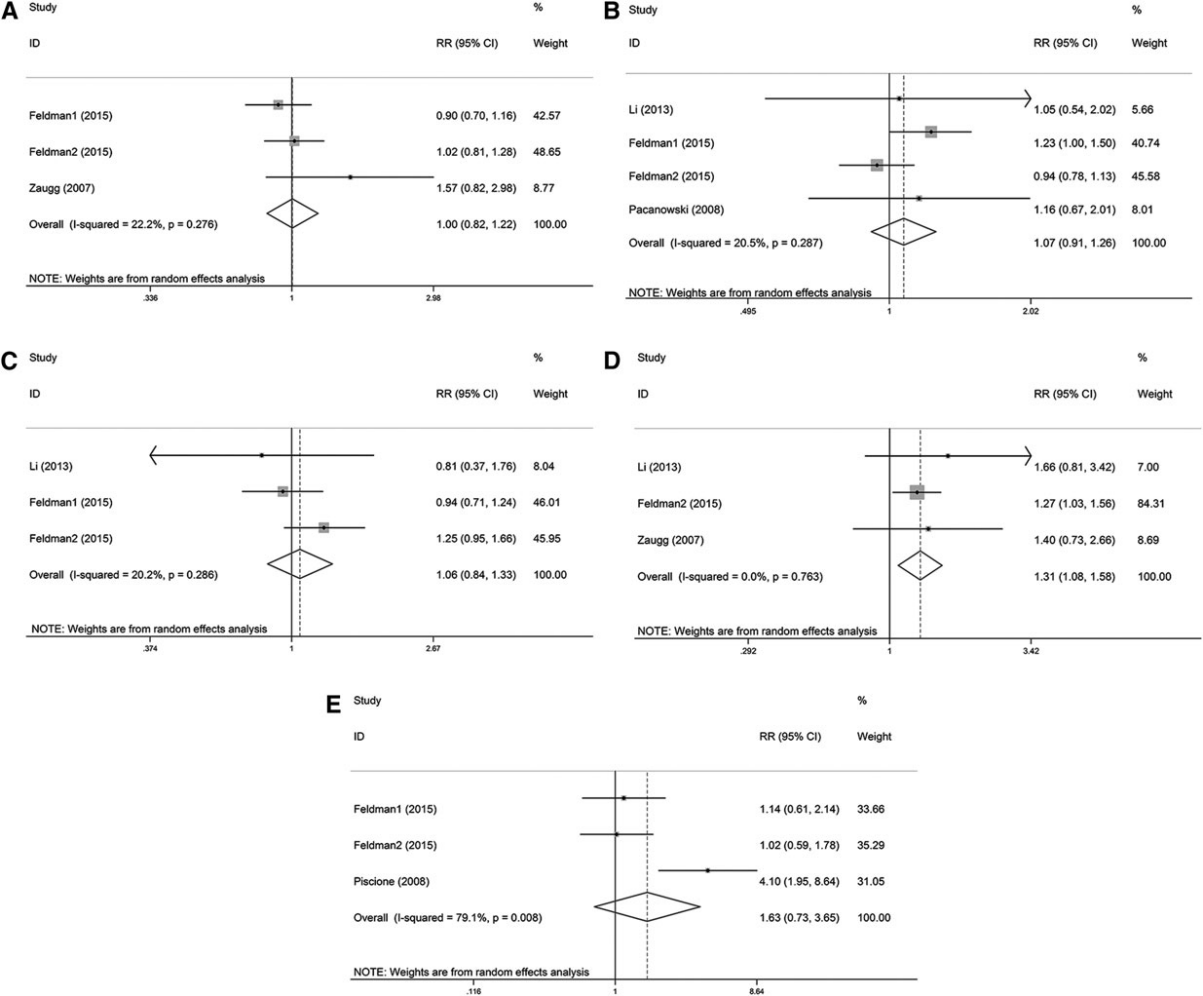
Forest plots of the association of *ADRB1* and *ADRB2* polymorphisms with cardiovascular events under the dominant model. **(A)** *ADRB1* rs1801252 polymorphism. **(B)** *ADRB1* rs1801253 polymorphism. **(C)** *ADRB2* rs1042713 polymorphism. **(D)** *ADRB2* rs1042714 polymorphism. **(E)** *ADRB2* rs1800888 polymorphism. CAD, coronary artery disease; CI, confidence interval; RR, risk ratio.

A positive association was found between the *ADRB2* rs1042714 polymorphism and cardiovascular events in CAD patients (RR = 1.31, 95% CI: 1.08–1.58, *p* = 0.006; Table 3 and Fig. 2D). Compared with patients who were Gln27 homozygotes, being a Glu27 carrier was associated with a 31% increase in the risk of cardiovascular events. No significant association was found between *ADRB1* (rs1801252, rs1801253), *ADRB2* (rs1042713, rs1800888) and cardiovascular events in CAD patients (Table 3 and Fig. 2A–C, E).

**Table 3.** Association of *ADRB1* and *ADRB2* Polymorphisms with Cardiovascular Events and All-Cause Mortality

*Outcome*	*Polymorphism*	*RR*	*95% CI*	p
Cardiovascular events	rs1801252	1.00	0.82–1.22	0.965
	rs1801253	1.07	0.91–1.26	0.389
	rs1042713	1.06	0.85–1.33	0.619
	rs1042714	1.31	1.08–1.58	0.006^*^
	rs1800888	1.63	0.73–3.65	0.234
All-cause mortality	rs1801252	0.95	0.79–1.13	0.534
	rs1801253	1.02	0.88–1.17	0.816
	rs1042713	1.21	0.85–1.73	0.295
	rs1042714	0.97	0.70–1.35	0.859
	rs1800888	1.36	0.78–2.35	0.281

^*^ *p* < 0.05.

**FIG. 3.**
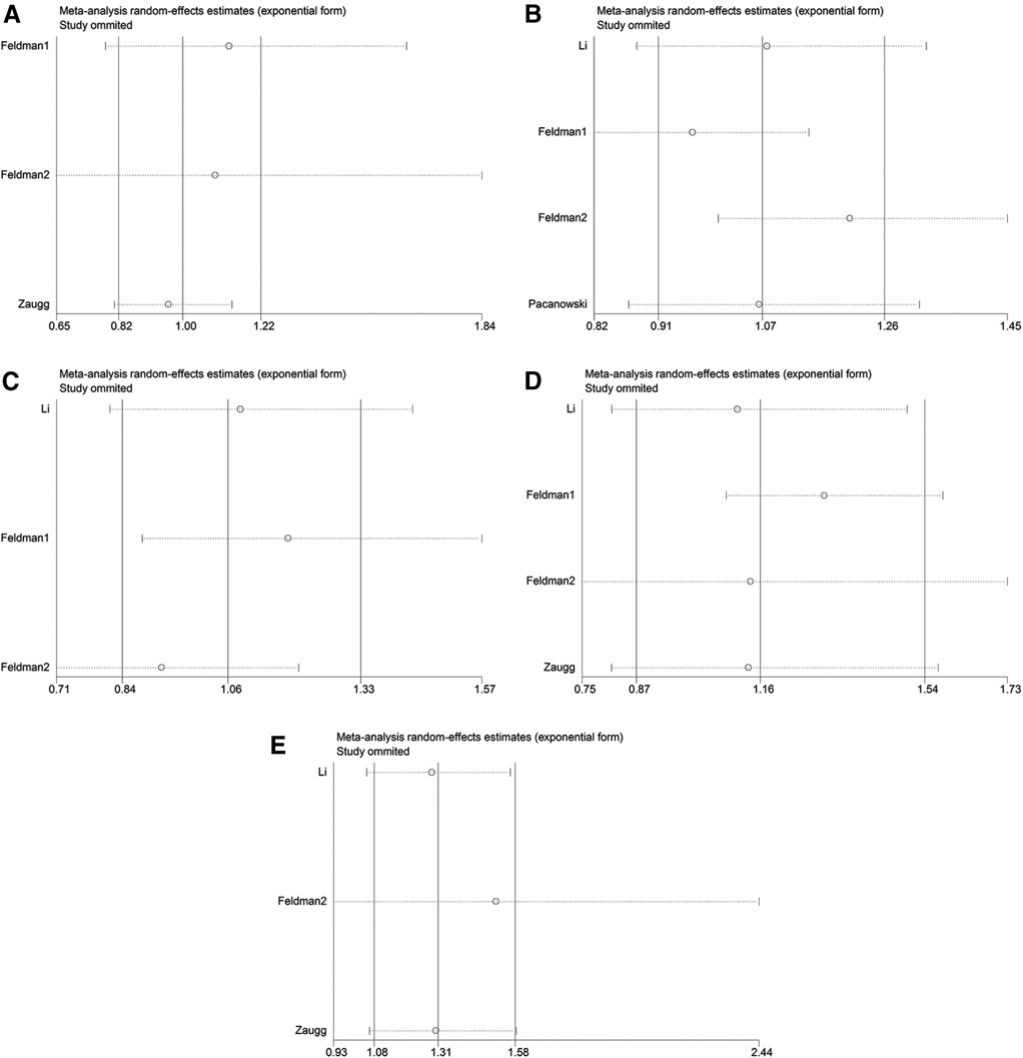
Sensitivity analyses of the association of *ADRB1* and *ADRB2* polymorphisms with cardiovascular events under the dominant model. **(A)** *ADRB1* rs1801252 polymorphism. **(B)** *ADRB1* rs1801253 polymorphism. **(C)** *ADRB2* rs1042713 polymorphism. **(D)** *ADRB2* rs1042714 polymorphism. **(E)** *ADRB2* rs1800888 polymorphism.

Significant heterogeneity was observed for the associations of *ADRB2* rs1042714 (*I*^2^ = 62.8%) and rs1800888 (*I*^2^ = 79.1%) with cardiovascular events. We performed a random-effects analysis, followed by sensitivity analyses. The results of the sensitivity analyses demonstrated that data from the Feldman 1 study deviated from data from other studies with respect to the association of the *ADRB2* rs1042714 polymorphism and the cardiovascular events (Fig. 3D). After removing this study, *I*^2^ was reduced from 62.8% to 0% (*p* = 0.893). Therefore, we excluded this study from the analysis of the association of the *ADRB2* rs1042714 polymorphism with cardiovascular events. None of the other studies showed deviations with respect to the association of the *ADRB2* rs1800888 polymorphism with cardiovascular events in CAD patients (Fig. 3E).

### Association of *ADRB1* and *ADRB2* polymorphisms with all-cause mortality

**FIG. 4.**
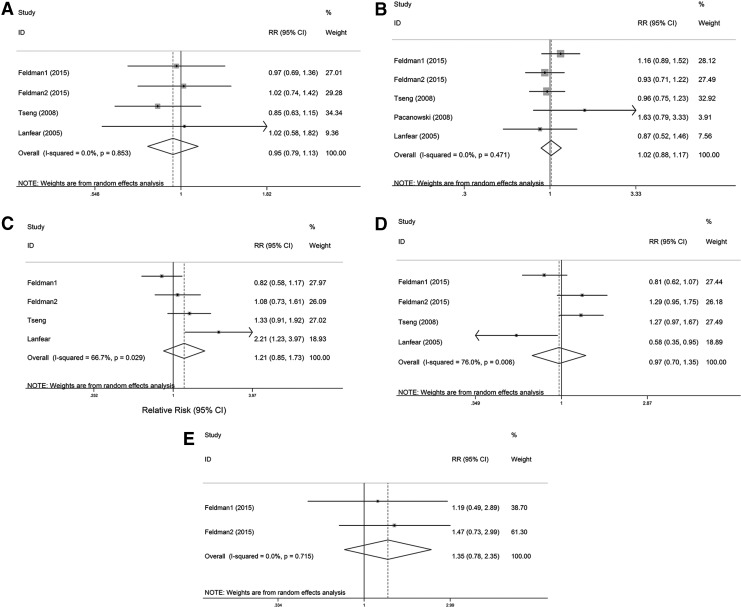
Forest plots of the association of *ADRB1* and *ADRB2* polymorphisms with all-cause mortality under the dominant model. **(A)** *ADRB1* rs1801252 polymorphism. **(B)** *ADRB1* rs1801253 polymorphism. **(C)** *ADRB2* rs1042713 polymorphism. **(D)** *ADRB2* rs1042714 polymorphism. **(E)** *ADRB2* rs1800888 polymorphism.

No significant associations were found between *ADRB1* (rs1801252 and rs1801253) or *ADRB2* (rs1042713, rs1042714, and rs1800888) polymorphisms and all-cause mortality in CAD patients (Table 3 and Fig. 4A–E).

**FIG. 5.**
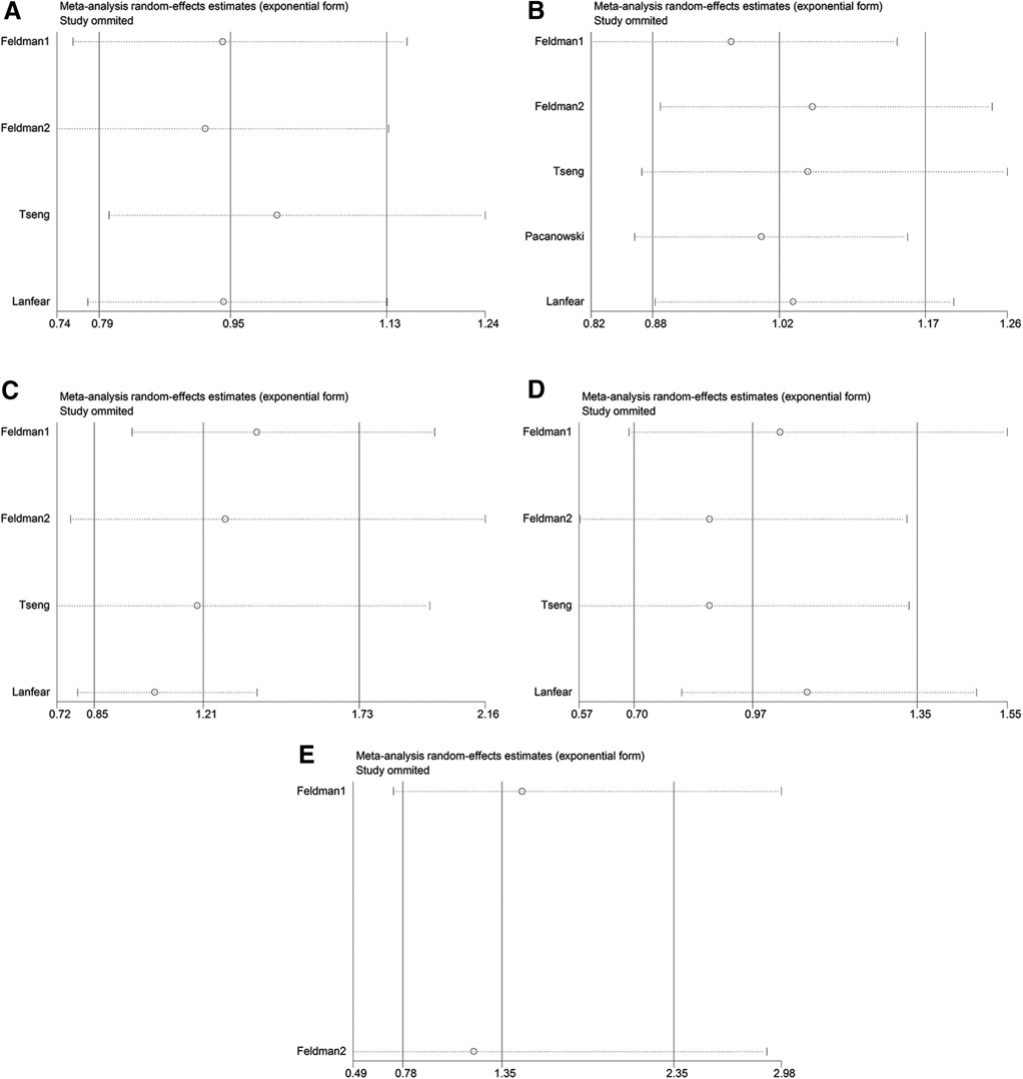
Sensitivity analyses of the association of *ADRB1* and *ADRB2* polymorphisms with and all-cause mortality under the dominant model. **(A)** *ADRB1* rs1801252 polymorphism. **(B)** *ADRB1* rs1801253 polymorphism. **(C)** *ADRB2* rs1042713 polymorphism. **(D)** *ADRB2* rs1042714 polymorphism. **(E)** *ADRB2* rs1800888 polymorphism.

Significant heterogeneity was found for the association of the *ADRB2* rs1042713 (*I*^2^ = 66.7%) and rs1042714 (*I*^2^ = 76.0%) polymorphism with all-cause mortality. Hence, a random-effects analysis was performed. We did not find any study that deviated from the other studies, as indicated by the sensitivity analyses (Fig. 5C, D).

### Publication bias

**FIG. 6.**
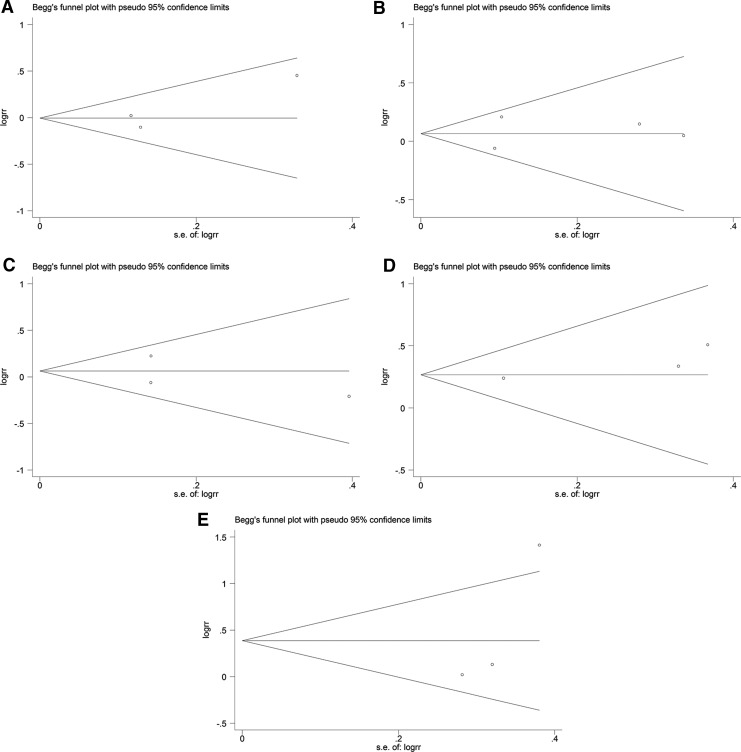
Begg's funnel plots of publication bias in the meta-analysis of the association of *ADRB1* and *ADRB2* polymorphisms with cardiovascular events under the dominant model. **(A)** *ADRB1* rs1801252 polymorphism. **(B)** *ADRB1* rs1801253 polymorphism. **(C)** *ADRB2* rs1042713 polymorphism. **(D)** *ADRB2* rs1042714 polymorphism. **(E)** *ADRB2* rs1800888 polymorphism. logrr, the logarithm of relative risk; s.e. of logrr, standard error of logrr.

**FIG. 7.**
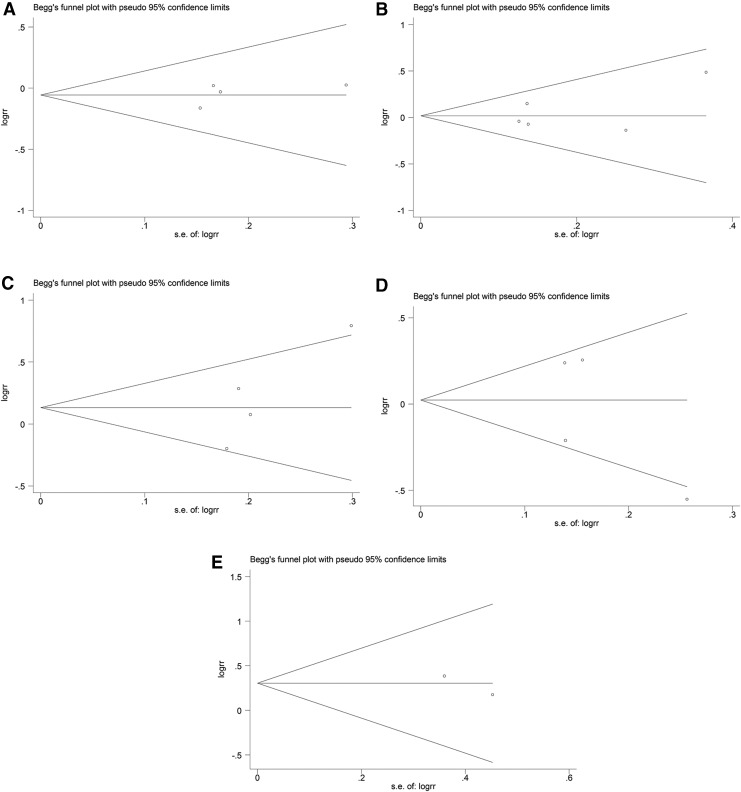
Begg's funnel plots of publication bias in the meta-analysis of the association of *ADRB1* and *ADRB2* polymorphisms with all-cause mortality under the dominant model. **(A)** *ADRB1* rs1801252 polymorphism. **(B)** *ADRB1* rs1801253 polymorphism. **(C)** *ADRB2* rs1042713 polymorphism. **(D)** *ADRB2* rs1042714 polymorphism. **(E)** *ADRB2* rs1800888 polymorphism. logrr, the logarithm of relative risk; s.e. of logrr, standard error of logrr.

**FIG. 8.**
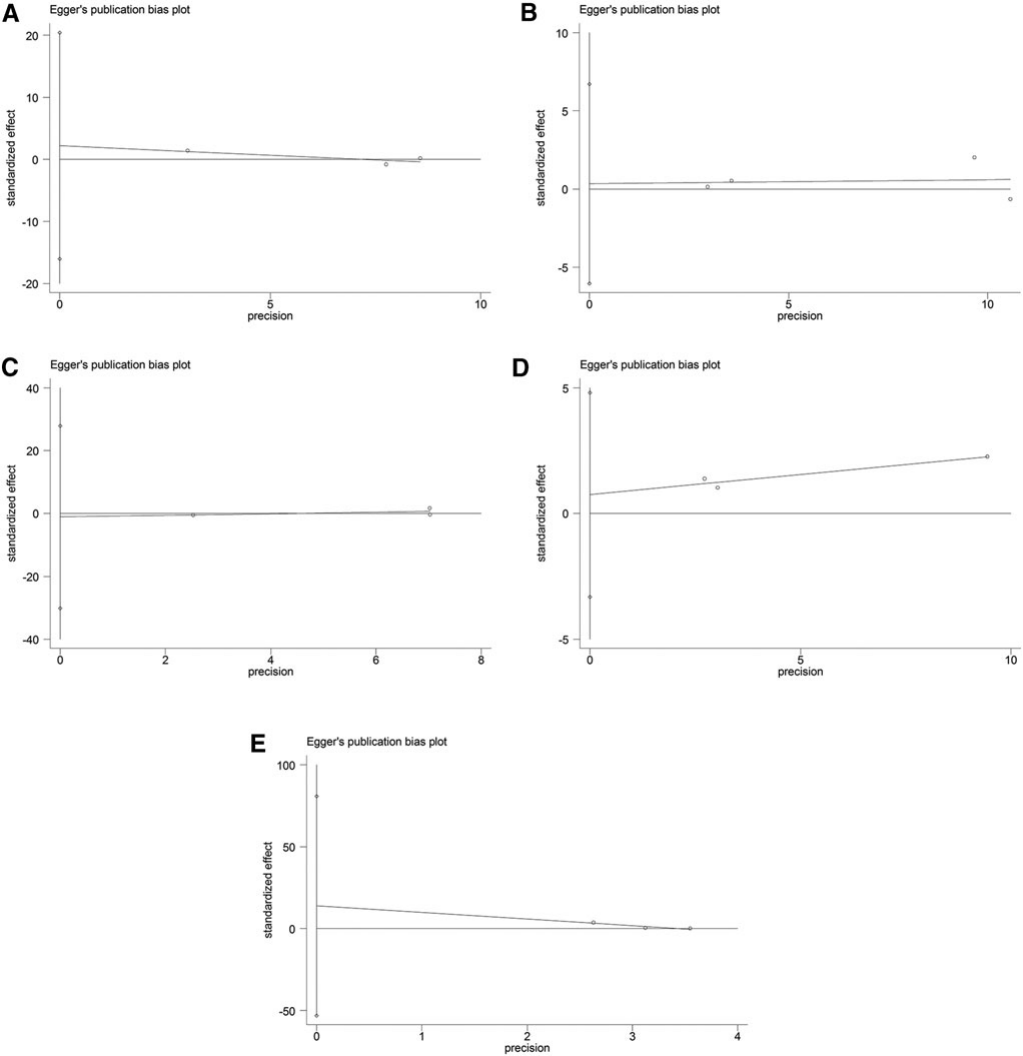
Egger's plots of publication bias in the meta-analysis of the association of *ADRB1* and *ADRB2* polymorphisms with cardiovascular events under the dominant model. **(A)** *ADRB1* rs1801252 polymorphism. **(B)** *ADRB1* rs1801253 polymorphism. **(C)** *ADRB2* rs1042713 polymorphism. **(D)** *ADRB2* rs1042714 polymorphism. **(E)** *ADRB2* rs1800888 polymorphism.

**FIG. 9.**
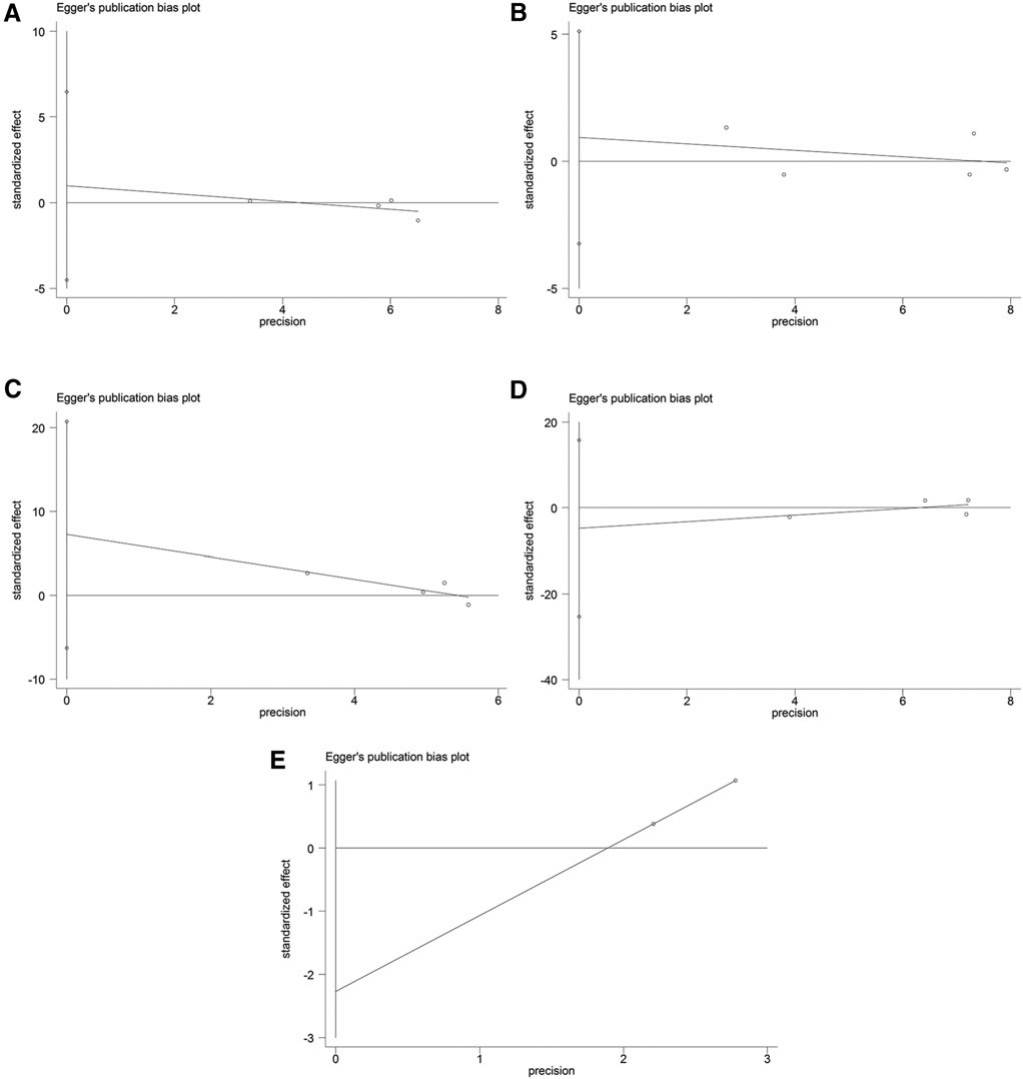
Egger's plots of publication bias in the meta-analysis of the association of *ADRB1* and *ADRB2* polymorphisms with all-cause mortality under the dominant model. **(A)** *ADRB1* rs1801252 polymorphism. **(B)** *ADRB1* rs1801253 polymorphism. **(C)** *ADRB2* rs1042713 polymorphism. **(D)** *ADRB2* rs1042714 polymorphism. **(E)** *ADRB2* rs1800888 polymorphism.

Begg's funnel plots (Figs. 6A–E and 7A–E) were generated, and Egger's tests (Figs. 8A–E and 9A–E) were performed to evaluate the potential publication bias. The shapes of the funnel plots showed no evidence of obvious asymmetry. The results of Egger's test did not support the existence of publication bias.

## Discussion

In the present meta-analysis, we examined whether specific genetic polymorphisms in the *ADRB1* and *ADRB2* genes were associated with cardiovascular events and all-cause mortality in CAD patients. To our knowledge, this was the first meta-analysis to explore the association of *ADRB1* and *ADRB2* polymorphisms with cardiovascular events and all-cause mortality in CAD patients, and the results suggested that *ADRB2* rs1042714 presented a positive association with cardiovascular events but not with all-cause mortality in CAD patients.

ADRB2 is expressed on coronary endothelial and vascular smooth muscle cells, which play an important role in the vasodilatation of the coronary arteries and microcirculation in normal coronary arteries (Barbato *et al.*, 2005; Hesse and Eisenach, 2008). Genetic polymorphisms of *ADRB2* have been reported to modulate the functional responses of the receptor to adrenergic stimulation (Dhein *et al.*, 2017), which may be associated with cardiovascular events and all-cause mortality in CAD patients. However, conflicting data regarding the association of the *ADRB2* rs1042714 polymorphism with cardiovascular events and all-cause mortality in CAD patients have been reported. Lanfear *et al.* (2005) demonstrated that Glu27 homozygosity at the *ADRB2* rs1042714 polymorphism was a protective factor for overall mortality in CAD patients treated with β-blockers. However, Tseng *et al.* (2008) demonstrated a trend toward increased mortality in Glu27 homozygotes compared to Gln27 carriers among postmenopausal women with CAD, and this finding was further confirmed by the surgical treatment for ischemic heart failure trials. Feldman *et al.* (2015) demonstrated that CAD patients harboring the Glu27 allele of the *ADRB2* rs1042714 polymorphism were at increased risk of mortality and cardiovascular events.

Our data demonstrated that Glu27 carriers at the *ADRB2* rs1042714 polymorphism exhibited an increased risk for cardiovascular events but not all-cause mortality. Potential reasons for this finding include the following: the *ADRB2* rs1042714 polymorphism results in the substitution of Glu for Gln at codon 27, and the “gain-of-function” of the receptor conferred by the Glu27 allele could cause target tissues to be overexposed to catecholamine, thus accelerating the development of CAD and exacerbating heart dysfunction (Barbato *et al.*, 2007). In addition, several studies have demonstrated an independent association of the Glu27 allele of the *ADRB2* rs1042714 polymorphism with a number of diseases, such as obesity, dyslipidemia, diabetes, and stroke (Kumar *et al.*, 2015). These disorders usually coexist with each other and could lead to development and progression of CAD (Jakovljevic and Ostojic, 2013). Lanfear *et al.* (2005) demonstrated that Glu27 homozygosity of the *ADRB2* rs1042714 polymorphism was a protective factor against all-cause mortality only in CAD patients who were treated with β-blockers, whereas they failed to demonstrate any protective effects in patients who were not treated with β-blockers. The reason could be that β-blockers that specifically target ADRB2 might attenuate adverse effects observed in Glu27 carriers at the *ADRB2* rs1042714 polymorphism (McLean *et al.*, 2011). Although we concluded that being a Glu27 carrier at the *ADRB2* rs1042714 polymorphism presented a positive association with cardiovascular events, we failed to observe this association for all-cause mortality in CAD patients. A possible explanation for this finding is that the influence of the *ADRB2* rs1042714 polymorphism on cardiovascular clinical outcomes is subtle and therefore might not increase the risk of all-cause mortality, but can still influence cardiovascular events.

No significant associations of the *ADRB1* (rs1801252, rs1801253) and *ADRB2* (rs1042713, rs1800888) polymorphisms with cardiovascular events and all-cause mortality in CAD patients were found. This result was in line with the conclusion of a large prospective cohort study that failed to find any association of the *ADRB1* rs1801253 and *ADRB2* rs1042713 polymorphisms with mortality under an additive model in CAD patients (Cresci *et al.*, 2012). Another study that examined the *ADRB2* rs1042713 and rs1800888 haplotype also found no association with revascularization and MI in patients with stable angina undergoing elective PCI (Rywik *et al.*, 2011).

### Limitations

Although our study was the first meta-analysis to address the association of *ADRB1* and *ADRB2* polymorphisms with cardiovascular events and all-cause mortality in CAD patients, it has some limitations. First, the number of studies involved in our meta-analysis was limited, which rendered the revealed associations less robust. Second, we could not obtain the specific cases and controls according to each genotype of the *ADRB1* and *ADRB2* polymorphisms; hence, we only calculated pooled RRs and 95% CIs under the dominant model. Third, we could not adjudicate causes of death although cardiovascular death is more likely to predominate. Because all-cause mortality instead of cardiovascular death was used for the primary outcome in most studies of this meta-analysis. Fourth, there were differences in the age, gender, and populations of the study cohorts as well as the inclusion and exclusion criteria, cardiovascular events, and duration of follow-up among these studies, which might account for the observed heterogeneity. In the study by Feldman *et al.*, patients with left ventricular dysfunction who present a different risk profile than the cohorts of other studies were enrolled.

In conclusion, this study suggests that *ADRB2* rs1042714 polymorphism might play a role in the prognosis of cardiovascular events and ultimately represent as an important genetic marker. CAD patients harboring the *ADRB2* rs1042714 polymorphism may need aggressive management to optimize their prognosis.
